# Management of stage III non-small-cell lung cancer: rays of hope

**DOI:** 10.37349/etat.2024.00206

**Published:** 2024-02-19

**Authors:** Floryane Kim, Maxime Borgeaud, Alfredo Addeo, Alex Friedlaender

**Affiliations:** Istituto Nazionale Tumori-IRCCS-Fondazione G. Pascale, Italy; ^1^Oncology Department, Geneva University Hospitals, 1205 Geneva, Switzerland; ^2^Oncology Department, Clinique Générale Beaulieu, 1206 Geneva, Switzerland

**Keywords:** Non-small-cell lung cancer, stage III, perioperative immunotherapy, targeted therapy, chemo-radiotherapy, multidisciplinary management

## Abstract

Lung cancer remains the most common cause of cancer death across the world. Non-small-cell lung cancer (NSCLC) represents the most frequent type of lung cancer and is frequently diagnosed at an advanced stage. Stage III NSCLC, which encompasses 30% of cases, refers to a state between localized and metastatic disease, and is associated with poor prognosis. As highlighted in this review, stage III represents a heterogenous group, whose complex management includes multimodal treatment, discussed below, and requires discussion in multidisciplinary teams. The goal of this approach is a maximalist attitude in these patients with locally advanced and non-metastatic disease. However, many issues remain under debate including the optimal sequences of treatment between different treatment modalities, patient selection particularly for surgery, the duration of perioperative treatments and the identification of biomarkers to determine which patients might benefit of specific treatment like immunotherapy and targeted therapies. This review describes the current landscape of management of stage III NSCLC, discussing the critical issue of resectability, and highlighting the recent advancements in the field, particularly the incorporation of immune-checkpoint inhibitors (ICIs) and targeted therapies in this setting.

## Introduction

Lung cancer is the leading cause of cancer-related death and can be divided into two main subgroups: small cell lung cancer and non-small-cell lung cancer (NSCLC) [1]. NSCLC accounts for more than 80% of diagnosed lung cancers and are divided into different histological subtypes which are represented mainly by adenocarcinoma, approximately 40% of cases, and squamous cell carcinoma, approximately 25–30% [2]. The majority of NSCLC are diagnosed at an advanced, regional or metastatic stage with poor outcomes [3, 4].

Locally advanced NSCLC is a heterogenous group and refers to a stage of lung cancer which has extended to nearby tissues, structures, or lymph nodes within the chest, but has not spread to distant organs or tissues. Typically, this corresponds to stage IIIA, IIIB and IIIC, according to the 8th and 9th classification of the tumor-node-metastasis (TNM) staging system [5]. It is a state between localized and metastatic lung cancer and requires a balance between local control and systemic management. The treatment approach for stage III NSCLC depends on tumor factors such as location and nodal involvement, patient characteristics, and surgical experience. Treatment is usually multimodal, including various combinations of therapy including surgery, radiotherapy (RT), and systemic treatment, and must be discussed in a multidisciplinary team [6]. A cornerstone in the management of stage III NSCLC is thus the determination of resectability. Stage III NSCLC can be classified as resectable, borderline resectable or unresectable. The aim of this review is to synthesize the latest advances for locally advanced NSCLC.

## Management of stage III NSCLC

In the following section, the treatment options of locally advanced NSCLC are discussed. To do so, the concept of resectability and treatment discussions are explored. Another important factor is the impact of oncogenic driver mutations and the growing role these will play in the near future. The most important trials to date are summarized in Table 1.

**Table 1:** Selected phase III trials

**Trial**	**Number of patients/characteristics**	**Intervention**	**Histologies (SCC/adeno)**	**Results**	**Main conclusion of the study**
CRT followed by surgery *vs.* definitive RT					
NCT00002550, 2009 [10]	*N* = 429 pts Stage IIIA (T1–3, pN2) Stage IIIB excluded	Arm 1: CRT and surgery Arm 2: CRT and definitive RT CT (cis-etoposide)	30%/38%	5-year PFS 22.4% (arm 1) *vs.* 11.1% mOS 23.6 months (arm 1) *vs.* 22.2 months, HR: 0.87, NS	No significant survival advantage of surgery compared to RT-CT
Induction CT, RT-CT followed by surgery *vs.* RT-CT					
ESPATUE, 2015 [11]	*N* = 161 pts, potentially resectable Stage IIIA (34%) Stage IIIB (65%) TNM 6th	Induction CT (×3 cis-paclitaxel) followed by CRT (cis-vinorelbin) and if resectable: CRT *vs.* surgery (arm1/2)	39%/47%	No difference in 5-year OS or PFS	No significant survival advantage of adding surgery to CT and RT-CT, but underpowered trial
CT before and after RT					
PROCLAIM, 2016 [16]	*N* = 598 pts, unresectable Stage IIIA (47%) Stage IIIB (52%) TNM 6th	Arm 1: CT (cis-pem) ×3 + TRT followed by pem ×4 Arm 2: CT (cis-etoposide) ×2 + TRT followed by platinum-based CT	-/75%	No difference in OS	Cis-etoposide or cis-pem can be considered with concomitant RT for adenocarcinoma
Adjuvant immunotherapy post-RT-CT					
PACIFIC, 2022 [17, 18]	*N* = 713 pts, unresectable Stage IIIA (53%) Stage IIIB (44%)	Durvalumab *vs.* placebo after ≥ 2 cycles of CRT without progression, for 12 months	46%/54%	5-year OS 42.9% for durvalumab *vs.* 33.4%, HR: 0.72 5-year PFS 33.1% *vs.* 19%, HR: 0.55	Significant improvement in OS/PFS with durvalumab post CRT
Adjuvant immunotherapy					
IMpower010, 2023 [26]	*N* = 1,005 pts, completely resected Stage II (52%) Stage IIIA (48%) PD-L1 ≥ 50% (23%) TNM 7th Adjuvant platinum-based CT	Adjuvant: atezolizumab for 1 year *vs.* BSC	40%/48%	mOS not estimable in ITT population, but trend shown in PD-L1 ≥ 50%, HR: 0.43	Significant improvement in DFS with atezolizumab post-surgery, regardless of PD-L1 status
PEARLS/Keynote-091, 2022 [25]	*N* = 1178 pts, completely resected Stade IB (14%), stage II (57%), stage IIIA (29%) TNM 7th PD-L1 TPS < 1% (39%), 1–49% (32%), ≥ 50% (28%) Adjuvant CT mandatory for stage II/IIIA	Adjuvant: pembrolizumab *vs.* placebo for 1 year	35%/64%	mDFS 53.6 months for pembrolizumab group *vs.* 42 months, HR: 0.76 mOS not reached in either group	Improvement of DFS with adjuvant pembrolizumab regardless of PD-L1 expression No requirement for EGFR/ALK testing (limitation)
Adjuvant targeted therapies					
ADAURA, 2023 [41, 42]	*N* = 682 pts, completely resected, EGFR positive Stage IB (32%), II (34%), IIIA (35%) Adjuvant CT recommended for stage II–IIIA	Osimertinib adjuvant for 3 years *vs.* placebo	-/97%	5-year OS 88% in the osimertimib group *vs.* 78%, HR: 0.49	Adjuvant osimertinib improves DFS and OS compared to placebo, in resected EGFR mutated NSCLC
ALINA, 2023 [44]	*N* = 257 pts, completely resected, ALK positive Stage IB–IIIA	Alectinib adjuvant for 2 years *vs.* CT	NA	mDFS: NR *vs.* 44.4 months in stage II–IIIA, HR: 0.24	Adjuvant alectinib improves DFS compared to adjuvant platinum-based CT, in resected EGFR mutated NSCLC
Neo-adjuvant CT-immunotherapy					
CheckMate-816, 2022 [30]	*N* = 773 pts, resectable Stage IB or II (35%) Stage IIIA (64%) TNM 7th PD-L1 < 1% (43%), 1–49% (27%), ≥ 50% (22%)	Platinum-based CT +/– nivolumab ×3, followed by surgery	50%/49%	mEFS 31.6 months in nivolumab group *vs.* 20.8 months, HR: 0.63 pCR: 24% in the nivolumab group *vs.* 2.2%	Significant improvement in EFS and pCR with neoadjuvant CT + nivolumab No higher incidence/greater severity of AE with nivolumab
Perioperative CT-immunotherapy					
Keynote-671, 2023 [32, 33]	*N* = 797 pts, resectable Stage II–IIIB	Cis-based CT + pembrolizumab *vs.* placebo for 4 neoadjuvant cycles, followed by surgery and adjuvant pembrolizumab *vs.* placebo for 1 year	NA	36-months EFS 54.3% in the pembrolizumab group *vs.* 35.4% 36-months OS 71.3% *vs.* 64%, HR 0.72. pCR 18.1% *vs.* 4%	Perioperative pembrolizumab improves OS, EFS and pathological response
CheckMate-77T, 2023 [31]	*N* = 461 pts, resectable Stage II–IIIB	Platinum-based CT + nivolumab *vs.* placebo for 4 neoadjuvant cycles, followed by surgery and adjuvant nivolumab *vs.* placebo for 1 year	51%/49%	mEFS NR *vs.* 18.4 months, HR: 0.58 pCR 25.3% *vs.* 4.7% MPR 35.4% *vs.* 12.1%	Perioperative immunotherapy improves EFS and pathological response
AEGEAN, 2023 [34, 35]	*N* = 802 pts, resectable Stage II–IIIB	Platinum-based CT + durvalumab *vs.* placebo for 4 neoadjuvant cycles, followed by surgery and adjuvant durvalumab *vs.* placebo for 1 year	46.2%/53%	mEFS in mITT: NR *vs.* 25.9 months. HR: 0.68 pCR: 17.2% *vs.* 4.3%. Difference in pCR: 13.0%	Perioperative immunotherapy improves EFS and pathological response
NEOTORCH, 2023 [36]	*N* = 404 pts, resectable Stage III	Platinum-based CT + toripalimab or placebo for 3 neoadjuvant cycles Followed by surgery and platinum-based CT + toripalimab or placebo for 1 adjuvant cycles Followed by toripalimab *vs.* placebo) for 12 adjuvant cycles	77%/32%	mEFS: NR *vs.* 15.1 months. HR: 0.40 MPR 48.5% *vs.* 8.4%	Perioperative immunotherapy improves EFS and pathological response

CRT: chemo-RT; CT: chemotherapy; Adeno: adenocarcinoma; SCC: squamous cell carcinoma; AE: adverse event; BSC: best supportive care; Cis: cisplatin; EFS: event-free survival; HR: hazard ratio; NS: not significative; nSCC: non-squamous cell carcinoma; OS: overall survival; pCR: pathological complete response; Pem: pemetrexed; PFS: progression-free survival; pts: patients; TRT: thoracic radiation therapy; DFS: disease-free survival; MPR: major pathological response; PD-L1: programmed cell death ligand-1; EGFR: epidermal growth factor receptor; ALK: anaplastic lymphoma kinase; mDFS: median disease-free survival; pN2: pathologic N2 stage; mOS: median overall survival; mEFS: median event-free survival; NR: not reported; ITT: intention to treat; mITT: modified intention to treat; TPS: tissue proportion score; NA: not available; + or –: with or without

### Definition of resectability and borderline resectable stage III NSCLC

As mentioned above, the definition of resectability is not universal, and practice varies across different centers and according to surgeon expertise. Across the variety of stage III disease however, some tumors are clearly resectable, while others are unequivocally unresectable. Cases that are upfront resectable consist mainly in T3 N1 tumors, and some T4 N0 or N1 disease. On the other side of the spectrum, N3 tumors are considered unresectable, as are most cases of clinically evident multi-station N2 or bulky N2 diseases. In between stands the case of stage IIIA tumors with single station, non-bulky clinically positive N2, for which surgical experience is crucial. Clinical practice varies by country, and guidelines differ on that topic [7]. In these situations, the benefit of surgical resection over CRT remains uncertain. Several trials have compared surgical with radiotherapeutic approaches. The EORTC-08941 [8] and NTOG [9] trials compared surgery *vs.* RT, after initial neoadjuvant CT for all patients, and showed no differences in OS or EFS. Importantly, in these trials a significant part of the patients attributed to surgery did not undergo complete R0 resection. Moreover, the sequential strategy of CT and RT used in these trials is known to be inferior to concurent CRT. Two additional phase III trials, INT013917 and ESPATUE, evaluated concurrent CRT with or without surgery. The INT0139 trial compared induction CRT followed by surgery *vs.* CRT followed by further RT [10]. A slight improvement in PFS was observed in favor of surgery (12.8 months *vs.* 10.5 months) with no difference in OS, a discrepancy potentially attributable to an excess mortality rate after pneumonectomy. In the ESPATUE trial, no OS or PFS difference was observed between induction CT followed by CRT and by surgery, *vs.* induction CT and CRT alone [11]. However, the trial closed prematurely, resulting in insufficient statistical power for the primary outcome.

These data and the resulting uncertainties underly the paramount importance of discussing these challenging cases in multidisciplinary tumorboard, with thoracic surgeons who are experienced in the management of these patients. In comparison to the 8th edition of the TNM staging system, the 9th edition, recently presented at the World Conference on Lung Cancer, redefined the mediastinal N stages, distinguishing between single ipsilateral N2a and multi-station ipsilateral N2b, following the observation of different prognosis in these subgroups [12]. However, the treatment choice will not be based solely on this new TNM staging, and resectability, should be determined at a multidisciplinary team discussion.

### Unresectable stage III NSCLC

For patients presenting with unresectable stage III NSCLC, the mainstay of management is definitive concurrent CRT, with a platinum-based CT doublet. Several trials and meta-analyses demonstrated the superiority of CRT over sequential CT and RT [13] with a five-year OS of 16%. The preferred CT regimens in this situation are cisplatin-etoposide [14] and carboplatin-paclitaxel [15], as well as cisplatin-pemetrexed for non-squamous histology [16].

The management of unresectable NSCLC has changed since the results of the PACIFIC trial. This double-blind, randomized, placebo-controlled trial evaluated the efficacy and safety of 12 months of adjuvant durvalumab, an immune-checkpoint inhibitor (ICI) targeting PD-L1, after definitive CRT with a platinum-based regimen, for unresectable NSCLC. The co-primary endpoints were PFS and OS, and the secondary endpoints included PFS at 12 months and 18 months, objective response rate (ORR), duration of response and safety, among others. Seven hundred and thirteen patients were randomized 2:1, to receive durvalumab 10 mg/kg every 2 weeks for up to 12 months, or placebo [17]. The five-year survival outcomes, published in 2021, with a median follow-up of 34.2 months, demonstrated a sustained benefit with a median OS of 47.5 months in the durvalumab arm *vs.* 29.1 months [HR: 0.72, 95% confidence interval (CI): 0.59–0.89] in the placebo arm and a median PFS of 16.9 months in the immunotherapy arm *vs.* 5.6 months (HR: 0.55, 95% CI: 0.45–0.68). This benefit was observed in the majority of prespecified subgroups with the notable exception of the EGFR positive subgroup (HR: 0.84, 95% CI: 0.40–1.75) [18]. However, these exploratory analyzes and the small size of this group require in-depth analyzes to confirm this trend. The ORR was better with durvalumab than placebo (28.4% *vs.* 16%, *P* < 0.001). Since these results, the PACIFIC regimen became standard of care for unresectable stage III NSCLC [19]. Like in advanced NSCLC, the optimal duration of durvalumab is unknown [20].

Oncogene addicted lung adenocarcinoma represents a subset of NSCLC patients that have worse response rates to ICI [21]. EGFR represents the most frequent oncogene in NSCLC. There are limited data of specific treatment for EGFR mutated patients in this setting. The LAURA trial, which is in progress (NCT03521154), is a randomized phase III, double-blind, placebo-controlled, that is currently assessing the efficacy and safety for osimertinib, a third-generation irreversible oral EGFR-tyrosine kinase inhibitor, as a maintenance for unresectable NSCLC after CRT, with exon 19 deletion (ex19del) or Leu858Ar (L858R) EGFR mutations [22]. Patients are randomized 2:1 to either osimertinib 80 mg once a day or placebo, until disease progression. The primary endpoint is PFS and the secondary endpoints are among others: central nervous system PFS and cumulative incidence at 12 months and 24 months, OS, PFS by mutation status, response/disease control rate and safety. The results are expected at the end of 2023.

### Resectable NSCLC

For stage III NSCLC that are deemed resectable upfront, the standard of care has long been surgery followed by adjuvant CT with a cisplatin doublet [23]. The commonly used regimens are cisplatin-vinorelbine, etoposide, docetaxel, pemetrexed or gemcitabine. Adjuvant CT increases OS by 5.4% at 5 years in NSCLC, for tumor of more than 4 cm or with nodal disease [24]. Recently, the major advance in the treatment landscape of resectable NSCLC is the incorporation of ICI with anti-programmed cell death 1 (anti-PD-1)/PD-L1 in the treatment sequence of these patients.

Results from the Keynote-091/PEARLS trial showed that adjuvant pembrolizumab improved DFS compared to a placebo for patients with resected stage IB–III NSCLC: DFS of 53.6 months *vs.* 42.0 months (HR: 0.76, 95% CI: 0.63–0.91, *P* = 0.0014) [25]. The IMpower010 trial showed comparable results for atezolizumab, which conferred a DFS benefit *vs.* observation for stage II–IIIA PD-L1 positive tumors: (HR: 0.66, 95% CI: 0.50–0.88, *P* = 0.0039) [26]. The latest update of IMpower010 also revealed an OS advantage in the PD-L1 high subgroup exclusively. There are differences between Keynote-91 and IMpower010. First, Keynote-091 compared ICI to placebo while IMpower010 had an open-label design. Second, all patients in IMpower010 received adjuvant CT before receiving ICIs, while in Keynote-091 adjuvant CT was at the investigator’s discretion. Third, IMpower010 clearly showed a direct association between PD-L1 expression and the benefit of ICI, while Keynote-091 did not find a benefit among patients with PD-L1 > 50%, a finding that contradicts the known predictive value of PD-L1 in metastatic stages and a growing body of data suggesting a similar predictive role of PD-L1 in early-stage NSCLC. These trials led to the approval of atezolizumab for patients with PD-L1 > 50% by the European Medicines Agency (EMA), United Kingdom, and Canada.

Recently, the results of several randomized phase II and III trials of neoadjuvant and pre-operative ICI, combined with CT for resectable NSCLC, have been presented or published [27]. The theoretical advantage of a neoadjuvant approach stands in an enhance antitumor immune response as tumor antigens and lymph nodes are still in place [28, 29].

The first phase III data came from the CheckMate-816 trial, which compared three neoadjuvant cycles of nivolumab and CT with CT alone [30]. This is the only phase III trial that did not include an adjuvant phase. Nivolumab improved the coprimary endpoints of EFS, with an (HR: 0.63, 95% CI: 0.49–0.93), and pCR, at 24.0% (95% CI: 18.0–31.0) *vs.* 2.2% (95% CI: 0.6–5.6). CheckMate-77T, on the other hand, evaluated the use of peri-operative ICI in combination with CT. Treatment consisted in 4 cycles of nivolumab-CT or placebo-CT, followed by surgery and adjuvant treatment of nivolumab 480 mg every four weeks or placebo for one year in total [31]. The primary endpoint of EFS was met, with a median EFS not reached *vs.* 18.4 months, and a HR for EFS of 0.58 (97.36% CI: 0.42–0.81; *P* = 0.00025). The Keynote-671 trial compared perioperative pembrolizumab and CT to neoadjuvant CT alone in resectable stage II–IIIB NSCLC, according to the TNM American Joint Committee on Cancer (AJCC) 8th edition [32, 33]. There were 4 cycles of neoadjuvant CT with placebo or pembrolizumab, followed by 13 cycles of adjuvant placebo or pembrolizumab. After a median follow-up of 36.6 months, EFS was improved in the intervention arm with (HR: 0.59, 95% CI: 0.48–0.72). MPR, defined as 10% or less of viable tumor cells was improved at 30.2% *vs.* 11.1%, as was pCR at 18.1% *vs.* 4%. Most importantly, the Keynote-671 trial was the first phase III trial to demonstrate a survival benefit in favor of perioperative ICI with an OS of 71.3% *vs.* 64.0% at three years (HR: 0.72, 95% CI: 0.56–0.93; *P* = 0.00517). The pembrolizumab-combination led to slightly more adverse events, which did not impair the surgical resection rates in the intervention group, compared to the control arm. The phase III AGEAN trial evaluated the combination of durvalumab-CT *vs.* placebo-CT for four cycles in the preoperative setting, followed by adjuvant durvalumab or placebo for one year [34, 35]. The durvalumab combination improved EFS and pCR compared to placebo: EFS (HR: 0.68, 95% CI: 0.53–0.88, *P* = 0.003902), and pCR: 17.2% *vs.* 4.3%. Finally, the NEOTORCH phase III trial compared the association of toripalimab with CT to placebo-CT for three pre-operative and one post-operative cycle, followed by one year of toripalimab or placebo[36]. The primary endpoint, EFS among stage III patients, was met with a (HR: 0.40, 95% CI: 0.277–0.565, *P* < 0.0001). There was also higher MPR and pCR in the toripalimab arm: 48.5% *vs.* 8.4% and 24.8% *vs.* 1.0%, respectively.

These phases III studies did not only include stage III patients [37]. In subgroup analyses, stage III patients derived equal or greater benefit from immunotherapy than stage II patients. Two phase II studies specifically looked at resectable stage III diseases. First, the NADIM-2 trial randomized patients between nivolumab-CT and CT alone [38]. Patients with R0 resection in the intervention group received 6 months of adjuvant nivolumab. Nivolumab improved pCR at 37% *vs.* 7% in the control group. The secondary endpoints of PFS and OS were also significantly better with nivolumab. The Chinese TD-FOREKNOW phase II trial, comparing neoadjuvant camrelizumab-CT to CT alone, showed comparable results, with a pCR rate of 32.6% (95% CI: 19.1–48.5%) with camrelizumab *vs.* 8.9% (95% CI: 2.5–21.2%) for the control group [39].

Several questions remain regarding neoadjuvant ICI in NSCLC. First, the role of PD-L1 expression requires clarification. As in other setting in NSCLC, PD-L1 positive patients seem to derive more benefit from ICI than PD-L1 negative patients. For instance, in an exploratory subgroup analysis of CheckMate-816, no EFS benefit was seen in PD-L1 negative patients from the a ddition of nivolumab to CT, with a HR for EFS of 0.81 (95% CI: 0.48–1.36) [40]. The EMA approved neoadjuvant nivolumab exclusively for patients with PD-L1 positive disease based on this analysis. Second, the question of the optimal CT backbone remains uncertain. At present, it is difficult to establish whether the efficacy of ICI is impacted by a specific CT regimen. Third, the role of the adjuvant ICI after neoadjuvant therapy remains open for debate. While cross-comparing trials should be done with caution, the EFS rate at 2 years seems similar in the Keynote-671 and CheckMate-816 trials. To specifically address the role of the adjuvant therapy, further studies will be necessary. An interesting approach would be to randomize patients based on post-operative biomarkers such as circulating tumor DNA. Finally, a major limitation of these phase III trials is post-protocol drug access. In CheckMate-816 and Keynote-671, more than a third of patients in the control group who relapsed and required new systemic treatment did not receive ICI, which is standard of care in this situation.

Regarding oncogene-addicted NSCLC and the efficacy of ICI, some patients with EGFR or ALK were included in the phase III trials [32, 34]. As previously mentioned, the value of ICI for these patients is uncertain and the subgroup analyses from these trials have too few patients to draw any conclusions. There are greater expectations for targeted therapies in this setting. Osimertinib has recently been approved for the adjuvant treatment of NSCLC harboring classical EGFR mutations following the results of the ADAURA trial [41]. Compared to placebo, three years of adjuvant osimertinib improved DFS in stage IB–IIIA patients according to the AJCC 7th edition, with (HR: 0.20, 99% CI: 0.14–0.30, *P* < 0.001). The benefit for stage IIIA patients is greatest, with a HR for DFS of 0.12 (95% CI: 0.07–0.20). The OS results have recently been published, showing an improvement in survival in the stage II–IIIA population, with an OS of 88% in the osimertinib group *vs.* 78% in the placebo at five years (HR: 0.49, 95% CI: 0.33–0.73, *P* < 0.001) [42]. A major limitation in the interpretation of these OS results is the low cross-over rate to osimertinib at progression for patients in the control group, with only about 40% of patients with progressive disease in the control group receiving osimertinib, even though it represents the standard of care in this situation [43]. The potential role of osimertinib in the neoadjuvant setting is currently being explored in the phase III NeoADAURA for patients with resectable stage II to IIIB NSCLC harboring classical EGFR mutations.

The use of adjuvant alectinib, an ALK inhibitor, for NSCLC harboring ALK rearrangement has recently been reported in the ALINA trial [44]. The trial evaluated two-years of adjuvant alectinib compared to standard adjuvant CT for resected ALK positive NSCLC and showed a clear DFS benefit for alectinib in stage II to IIIA, with (HR: 0.24, 95% CI: 0.13–0.45). Several questions remained, however, such as if these impressive results will translate in an OS benefit such as in the ADAURA study; if adjuvant CT should still be considered for some patients in addition to alectinib; and if other ALK inhibitor such as lorlatinib could even do better in that setting [44].

As seen with EGFR, such trials have the potential to reshape the therapeutic sequence in other oncogenic alterations. As data mature, it may be difficult not to extrapolate the benefit to rarer oncogenic drivers for which organizing early-stage phase III randomized trials are unrealistic.

As highlighted by these new data on perioperative ICI and targeted therapies for stage III disease, the determination of resectability remains the cornerstone of the management of these patients, guiding towards a strategy of surgery or CRT. Recent data of pre- and perioperative ICI and the relatively high rate of MPR or pCR could challenge the definition of resectability, and question if stage IIIB disease should be directed toward surgery in priority. However, if the high rates of R0 resections of stage IIIB disease observed in trials involving highly experienced thoracic surgeons [38] can be extrapolated in real-life setting remains debated. Moreover, in all the phases III perioperative ICI trials, about 20% of patients were not able to undergo surgery, even if all included patients were judged resectable upfront. Thus, the rate of patients with R0 would certainly be much lower if patients with borderline or unresectable disease were included in a perioperative and resection strategy in real-life settings. Having to redirect patients with incomplete resection towards a salvage strategy could impose potential higher risks of toxicities, and possibly worst outcome than an upfront CRT strategy followed by durvalumab. Therefore, in situations where resectability is uncertain upfront, induction chemoimmunotherapy does not yet constitute a strategy that should be adopted unconditionally, at the present time.

## Conclusions

As we have discussed in this review, the management of stage III NSCLC is rapidly evolving, both in non-oncogene and oncogene-driven diseases. Though head-to-head trials have not been performed, given the growing wealth of data supporting a DFS and OS benefit, we expect a paradigm shift toward neoadjuvant and perioperative therapy in resectable disease. In inoperable NSCLC, immunotherapy consolidation after definitive CRT remains standard of care but the coming years may lead to combined immunotherapy approaches, as well as targeted therapies in oncogene-driven NSCLC. Current data should be used on a case-by-case basis and discussed in a multidisciplinary tumorboard to offer the best treatment plan to each patient.

## Abbreviations

ALK: anaplastic lymphoma kinase
CI: confidence interval
CRT: chemo-radiotherapy
CT: chemotherapy
DFS: disease-free survival
EFS: event-free survival
EGFR: epidermal growth factor receptor
HR: hazard ratio
ICI: immune-checkpoint inhibitor
mEFS: median event-free survival
mOS: median overall survival
MPR: major pathological response
NR: not reported
NSCLC: non-small-cell lung cancer
OS: overall survival
pCR: pathological complete response
PD-L1: programmed cell death ligand-1
PFS: progression-free survival
RT: radiotherapy
TNM: tumor-node-metastasis

## Declarations

### Copyright

© The Author(s) 2024.
